# Management of Patients With High Baseline Hip Fracture Risk by FRAX Reduces Hip Fractures—A Post Hoc Analysis of the SCOOP Study

**DOI:** 10.1002/jbmr.3411

**Published:** 2018-03-23

**Authors:** Eugene McCloskey, Helena Johansson, Nicholas C Harvey, Lee Shepstone, Elizabeth Lenaghan, Ric Fordham, Ian Harvey, Amanda Howe, Cyrus Cooper, Shane Clarke, Neil Gittoes, Alison Heawood, Richard Holland, Tarnya Marshall, Terence W O'Neill, Tim J Peters, Niamh Redmond, David Torgerson, John A Kanis, the SCOOP Study Team

**Affiliations:** ^1^ Mellanby Centre for Bone Research Centre for Integrated Research in Musculoskeletal Ageing University of Sheffield and Sheffield Teaching Hospitals Foundation Trust Sheffield UK; ^2^ Australian Catholic University Melbourne Australia; ^3^ MRC Lifecourse Epidemiology Unit and NIHR Southampton Nutrition Biomedical Research Centre University of Southampton and University Hospital Southampton NHS Foundation Trust Southampton UK; ^4^ Norwich Medical School University of East Anglia Norwich UK; ^5^ NIHR Musculoskeletal Biomedical Research Unit University of Oxford Oxford UK; ^6^ Department of Rheumatology University Hospitals Bristol Bristol UK; ^7^ Centre for Endocrinology Diabetes, and Metabolism Queen Elizabeth Hospital Birmingham UK; ^8^ Bristol Medical School University of Bristol Bristol UK; ^9^ Leicester Medical School Centre for Medicine University of Leicester Leicester UK; ^10^ Norfolk and Norwich University Hospital Norwich UK; ^11^ Arthritis Research UK Centre for Epidemiology Manchester Academic Health Science Centre The University of Manchester and NIHR Manchester Biomedical Research Centre Central Manchester University Hospitals NHS Foundation Trust Manchester UK; ^12^ Department of Health Sciences University of York York UK; ^13^ Centre for Metabolic Bone Diseases University of Sheffield, Sheffield, UK and Australian Catholic University Melbourne Australia

**Keywords:** HIP FRACTURE RISK, OSTEOPOROSIS, FRAX

## Abstract

The Screening for Osteoporosis in Older Women for the Prevention of Fracture (SCOOP) study was a community‐based screening intervention in women aged 70 to 85 years in the United Kingdom. In the screening arm, licensed osteoporosis treatments were recommended in women identified to be at high risk of hip fracture using the FRAX risk assessment tool (including bone mineral density measurement). In the control arm, standard care was provided. Screening led to a 28% reduction in hip fractures over 5 years. In this planned post hoc analysis, we wished to examine for interactions between screening effectiveness on fracture outcome (any, osteoporotic, and hip fractures) on the one hand and baseline FRAX 10‐year probability of hip fracture on the other. All analyses were conducted on an intention‐to‐treat basis, based on the group to which women were randomized, irrespective of whether screening was completed. Of 12,483 eligible participants, 6233 women were randomized to screening, with treatment recommended in 898 (14.4%). No evidence of an effect or interaction was observed for the outcomes of any fracture or osteoporotic fracture. In the screening arm, 54 fewer hip fractures were observed than in the control arm (164 versus 218, 2.6% versus 3.5%), and commensurate with treatment being targeted to those at highest hip fracture risk, the effect on hip fracture increased with baseline FRAX hip fracture probability (*p* = 0.021 for interaction); for example, at the 10th percentile of baseline FRAX hip probability (2.6%), there was no evidence that hip fractures were reduced (hazard ratio [HR] = 0.93; 95% confidence interval [CI] 0.71 to 1.23), but at the 90th percentile (16.6%), there was a 33% reduction (HR = 0.67; 95% CI 0.53 to 0.84). Prior fracture and parental history of hip fracture positively influenced screening effectiveness on hip fracture risk. We conclude that women at high risk of hip fracture based on FRAX probability are responsive to appropriate osteoporosis management. © 2018 The Authors. *Journal of Bone and Mineral Research* Published by Wiley Periodicals, Inc.

## Introduction

Over the last few years, treatment based on the absolute risk of fracture has been incorporated into many national and international guidelines, given the availability of validated fracture risk assessment tools such as FRAX, launched in 2008.^1^, ^2^ For example, in the United Kingdom, specific age‐dependent intervention thresholds based on 10‐year probability of fracture are advocated by the National Osteoporosis Guideline Group in their recent NICE‐accredited guidance.^3^ A cornerstone of the clinical utility of fracture risk tools is that the risk identified should be reversible by bone‐targeted therapies, and a number of post hoc analyses of phase 3 clinical trials have demonstrated that this is the case for risk identified by FRAX.^(4–11)^ Very recently, the first study that prospectively screened patients using FRAX probabilities to assess fracture risk (the Screening for Osteoporosis in Older Women for the Prevention of Fracture [SCOOP] study) has been published.^12^ This UK multicenter study, largely primary care based, assessed the effectiveness of a FRAX‐based, community screening program in women aged 70 to 85 years, with treatment targeted at women at high risk of hip fracture, compared with a control group receiving standard clinical care. The approach appears to be acceptable to both patients and GPs.^13^ During 5 years of follow‐up, prescriptions for anti‐osteoporosis medications were more frequent, and hip fracture incidence lower, in the screening intervention arm compared with the control arm (Table 1). Anti‐osteoporosis medications were particularly frequently prescribed in those intervention participants classified as high fracture risk, and so we hypothesized that the effect of screening to reduce hip fractures would be greatest in women with higher baseline FRAX probability, with a consequent interaction between baseline FRAX hip fracture probability and screening effectiveness.

**Table 1:** Fracture and Mortality Outcomes in the SCOOP Trial of Screening in Women Aged 70 to 85 Years in England^12^

Outcomes	Control (*n* = 6250)	Screening (*n* = 6233)	Hazard ratio^a^	*p* Value
OP‐related fractures	852 (13.6%)	805 (12.9%)	0.94 (0.85–1.03)	0.178
Any clinical fracture	1002 (16.0)	951 (15.3)	0.94 (0.86–1.03)	0.18
Hip fractures	218 (3.5%)	164 (2.6%)	0.72 (0.59–0.89)	0.002
Deaths	525 (8.4%)	550 (8.8%)	1.05 (0.93–1.19)	0.436

OP = osteoporosis (excludes fractures of the hands, feet, nose, skull, or cervical vertebrae).

^a^ Adjusted for recruiting region, baseline FRAX probability, and falls.

## Materials and Methods

The SCOOP clinical study was a pragmatic, unblinded, two‐group, parallel, randomized controlled trial to assess the effectiveness and cost‐effectiveness of screening to prevent fractures in older women; the design and results have been published previously.^12^, ^14^ In brief, women aged 70 to 85 years, not already on osteoporosis medications but suitable to participate, were approached through primary‐care lists in and around seven regions of England. Written, informed consent was obtained from all agreeing to participate, and they completed a self‐filled questionnaire capturing the FRAX risk factors before being randomized to the intervention (screening) or control arm. Baseline data comprised age, sex, height, and weight for body mass index (BMI) calculation and dichotomized risk variables including a prior fragility fracture since the age of 50 years, parental history of hip fracture, current tobacco smoking, any long‐term use of oral glucocorticoids, rheumatoid arthritis, other causes of secondary osteoporosis, and daily alcohol consumption of ≥3 units daily. If the respondent did not know the answer to an individual question, a negative response was assumed.

In the screening arm, the baseline risk factor questionnaire was used to calculate the 10‐year probability of hip fracture using the FRAX risk algorithm. Women deemed at moderate to high risk of hip fracture were invited to undergo a dual‐energy X‐ray absorptiometry (DXA) measurement of femoral neck bone mineral density (BMD) measurement and the 10‐year hip fracture probability was recalculated with inclusion of BMD. The final risk category (low or high) was communicated to the participant and family doctor by letter; participants remaining at high risk after incorporation of BMD into FRAX were advised to make an appointment with their family doctor to discuss treatment options.

In the control arm, apart from a letter to the general practitioner informing them of their patient participating in the study, no additional information was provided and they received usual care. The baseline 10‐year FRAX probabilities, without the inclusion of BMD, were calculated at the end of the trial for comparative purposes only.

During 5 years of follow‐up, exposure to osteoporosis treatment was higher in the screening arm with around 24% of the screening arm participants receiving at least one prescription for anti‐osteoporosis medication compared with 16% of the control arm. The difference reflected a high uptake of treatment in the high‐risk group within the screening arm with 703 women (78.3% of the high‐risk group) having received at least one prescription of anti‐osteoporosis medication within 6 months of randomization.

### Fracture outcomes

Only verified fractures at any anatomical site within the 5‐year follow‐up period were included as outcomes. In brief, we captured self‐reported fractures as well as searching routine Hospital Episode Statistics data, comprising information on hospital inpatient stays and emergency department attendance, together with primary‐care records to identify fractures in any of the study participants from the point of randomization until the end of follow‐up. Only independently confirmed fractures were included. Incident osteoporosis‐related fractures were defined as those excluding the hands, feet, nose, skull, or cervical vertebrae. Hip fractures were defined as verified fractures with a specific description of “neck of femur” or “proximal femur.”

### Statistical analysis

Because BMD measurements were not undertaken in all participants, the FRAX 10‐year hip fracture probability calculated without BMD was used in the analyses because this was available in both the control and intervention groups. A Poisson model was used to study the relationship between age, the time since baseline, invitation for screening, and FRAX 10‐year probability of hip fracture on the one hand, and on the other hand, the risk of any fracture, osteoporotic fracture or hip fracture with only one fracture being counted per patient during follow‐up (expressed as person‐years). A reduction in hip fracture risk that showed no interaction, with a flat reduction in risk across the range of baseline risk, would have suggested that factors other than treatment could have explained the observed effect.

All analyses were conducted on an intention‐to‐treat basis with participants analyzed according to the group to which they were randomized, irrespective of whether screening was completed. The hazard function was assumed to be exp(β_0_ + β_1 _· current time from baseline + β_2 _· current age + β_3 _· 10‐year probability + β_4 _· screening + β_5 _· 10‐year probability · screening). The beta coefficients reflect the importance of the variables as in a logistic model, and β_x_ = 0 denotes that the corresponding variable does not contribute to fracture risk. The variable “10‐year probability · screening” tested for an interaction between screening effectiveness and baseline 10‐year probability, handled as a continuous variable, by determining if β_5_< >0. Hazard ratios (HR) for screening effect and 95% confidence intervals (95% CI) were computed as a continuous variable. For presentation, hazard ratios were shown at the 10th, 25th, 50th, 75th, and 90th percentile of fracture probability. Further analyses explored the interaction between effectiveness and individual clinical risk factors within FRAX to determine the drivers of any potential interaction. A similar analysis was conducted for any fracture and all incident osteoporotic fractures. An exploratory analysis used the 10‐year probability of major osteoporotic fracture instead of hip fracture as the baseline risk.

## Results

### Baseline characteristics

A total of 12,483 women were randomized, with 6250 assigned to the control group and 6233 to the screening arm. The two groups were very similar at baseline (Table 2), with a mean age of 75 years and mean BMI just under 27 kg/m^2^ in both groups. The completion rates for the risk factors in the questionnaire were all above 90%, ranging from 92.6% for parental history of hip fracture to 99.9% for current smoking, and were similar in the screening and control arms. The prevalence of FRAX risk factors in the control group ranged from 3.6% for average alcohol intake above 3 units per day to 23.4% for prior fracture, with very similar rates in the screening arm. After DXA measurement of femoral neck BMD in 2817 women considered to be at moderate/high risk in the screening arm and recalculation of their FRAX hip fracture probabilities, 898 (14.4% of the screening arm) were identified to be at high risk and treatment recommended via their general practitioner. As expected, given the risk factors included in FRAX, these women tended to be older, have lower BMI, and a higher prevalence of risk factors than those in the control arm (Table 2). The mean FRAX 10‐year probability of hip fracture, calculated without BMD, was more than twofold higher in those identified at high risk compared with the control group.

**Table 2:** Baseline Characteristics of the Control and Screening Arms, Including Details of Those Identified at High Risk Within the Screening Group

	Control (*n* = 6250)	Screening (*n* = 6233)	Screened high risk (*n* = 898)
Age (years), mean (SD)	75.5 (4.1)	75.4 (4.2)	77.2 (4.4)
Body mass index, mean (SD)	26.7 (4.8)	26.7 (4.7)	24.4 (4.1)
Self‐reported prevalence, *n* (%)			
Fracture since age 50 years	1463 (23.4%)	1399 (22.4%)	409 (46.0%)
Parental hip fracture	577 (9.2%)	585 (9.4%)	354 (41.6%)
Smoking	290 (4.6%)	290 (4.7%)	86 (9.6%)
Alcohol ≥3 units/d	225 (3.6%)	219 (3.5%)	60 (6.7%)
Glucocorticoid use	312 (5.0%)	316 (5.1%)	113 (13.3%)
Rheumatoid arthritis	410 (6.6%)	426 (6.8%)	79 (9.3%)
Secondary causes of OP	1408 (22.5%)	1483 (23.8%)	267 (29.7%)
FRAX 10‐year hip fracture probability (without BMD); mean (SD)	8.5% (7.3%)	8.5% (7.4%)	17.9% (10.9%)

OP = osteoporosis/osteoporotic.

### Baseline FRAX hip fracture probability and observed incidence of fractures

**Figure 1:**
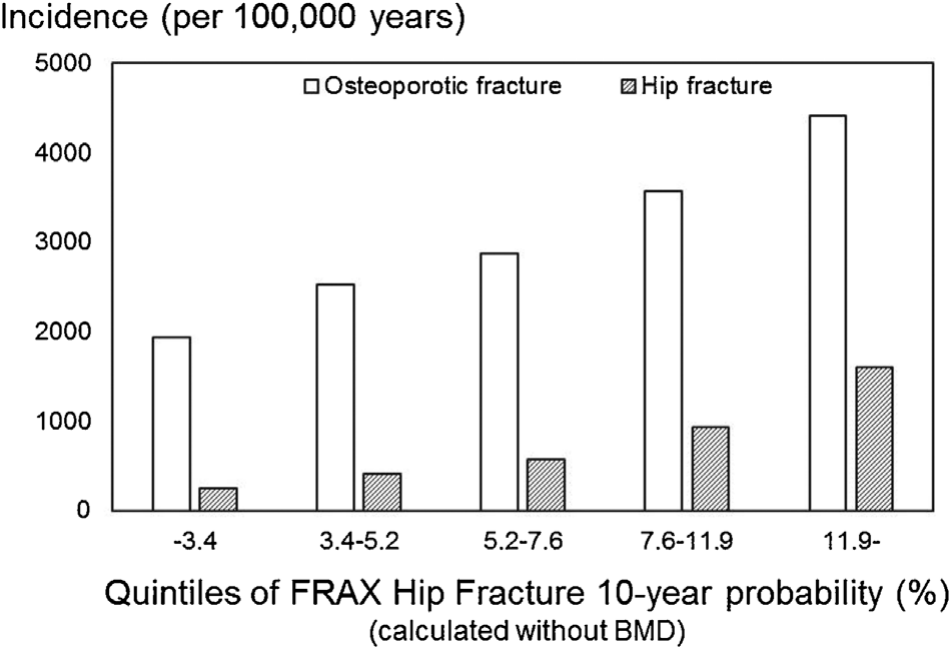
Observed incidence of osteoporotic and hip fractures during follow‐up in the control arm of the SCOOP study, within quintiles of baseline FRAX hip probability.

Over 5 years of follow‐up, 3.5%, 11.6%, and 13.6% of the control arm sustained a new hip fracture, a new major osteoporotic fracture, or any new osteoporotic fracture, respectively. There was evidence of an increase in the observed incidence of hip and osteoporotic fractures in the control arm of the study (Fig. 1) across the range of baseline FRAX hip fracture probability. For example, the observed incidence of hip fracture was 6.5‐fold higher in the highest quintile of baseline risk compared with the lowest quintile of risk. The incidence of osteoporotic fractures also increased in a stepwise fashion across the quintiles of risk, but the increase was less marked than that found in hip fractures, with only a 2.3‐fold increase from lowest to highest quintile.

### Effectiveness and relationship to FRAX

In Table 3, the effects of screening on various categories of fracture outcomes are shown, according to the baseline FRAX 10‐year probability of a hip fracture. The latter was entered as a continuous variable in the model, but for illustrative purposes, the table shows the effect at various values of baseline probability. Confidence estimates for the hazard ratio crossed unity at all probabilities for any fracture and osteoporotic fractures, with no evidence of an interaction between effectiveness and baseline FRAX hip fracture probability.

**Table 3:** Hazard Ratio (95% Confidence Interval) Between Screening and Control Arms for any Fracture, Osteoporotic Fracture, and Hip Fracture at Different Values of FRAX 10‐Year Probability (%) of a Hip Fracture Calculated Without Bone Mineral Density

Centile	FRAX probability	Any fracture	Osteoporotic fracture	Hip fracture
10th	2.6	0.96 (0.86–1.08)	0.97 (0.85–1.09)	0.93 (0.71–1.23)
25th	3.8	0.96 (0.86–1.07)	0.96 (0.86–1.08)	0.91 (0.70–1.17)
50th	6.3	0.96 (0.87–1.05)	0.96 (0.86–1.06)	0.85 (0.68–1.08)
75th	10.5	0.95 (0.87–1.04)	0.95 (0.86–1.04)	0.77 (0.63–0.95)
90th	16.8	0.94 (0.84–1.05)	0.93 (0.83–1.05)	0.67 (0.53–0.84)
*p* Value		>0.30	>0.30	0.021

The *p* value is for the interaction between screening and the outcome.

**Figure 2:**
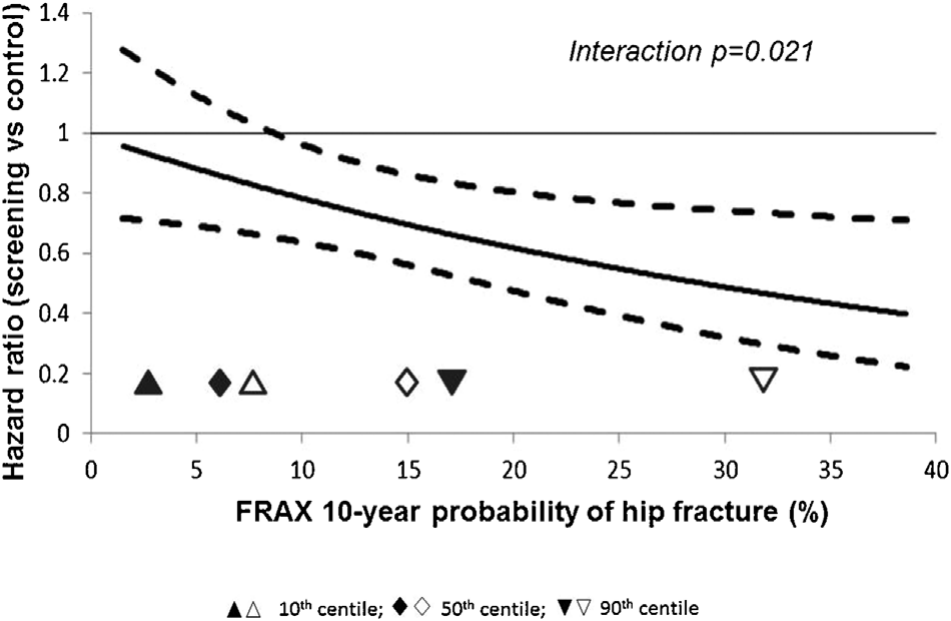
Impact of screening on hip fracture compared with control arm, expressed as hazard ratio, across range of FRAX 10‐year hip fracture probabilities at baseline, calculated without BMD. There was evidence of an interaction of effectiveness with baseline probability (*p* = 0.021). The symbols indicate the range of baseline probabilities in the whole study population (closed symbols) and in the high‐risk group identified by screening (open symbols).

In contrast, the hazard ratio showed an interaction with baseline FRAX hip fracture probability for the outcome of hip fracture (*p *= 0.021), and the upper confidence intervals were below unity at higher baseline risk (Table 3). The interaction between screening effect and fracture probability for the outcome of hip fracture is shown in Fig. 2. At the median value of baseline hip fracture probability in the whole study population (6.3%), invitation for screening was associated with a 15% reduction in hip fracture risk albeit with a 95% CI that included the null. At the 90th centile of the whole population risk (16.8%), there was a larger 33% reduction with a 95% CI that excluded the null, and at the 90th centile of baseline risk in the high‐risk population (32%), the reduction was even higher at 53% (95% CI 26% to 70%) (Fig. 2).

### Components of screening effect

In the context of evidence for an interaction between fracture probability and screening, we explored the potential drivers of the screening effect by examining the effect on hip fracture risk as a function of each variable within FRAX. For the continuous variables of age and BMI, there was no evidence of an interaction with screening. For example, at a BMI of 21 kg/m^2^, screening reduced hip fracture risk by 24% (HR = 0.76; 95% CI 0.57 to 1.01), with a similar reduction at a BMI of 33 kg/m^2^ (HR = 0.73; 95% CI 0.47 to 1.14). BMD could not be included in this analysis because of its absence in the vast majority of women in the control arm.

The interaction between screening effectiveness on hip fracture risk and the dichotomous variables within FRAX are shown in Table 4. For both prior fracture and parental history of hip fracture, there was evidence of an interaction, with greater effect on hip fracture reduction in those with the risk factor present. In contrast, an interaction was observed for prevalent smoking whereby non‐smokers appeared to achieve the benefit, whereas current smokers did not.

**Table 4:** Hazard Ratio Between Study Arms (Screening Versus Control) for Hip Fractures in Those With and Without the Presence of a Risk Factor at Baseline

	Hazard ratio (95% confidence interval)		
FRAX variable	Absent	Present	*p* Value
Previous fracture	0.87 (0.68–1.12)	0.55 (0.38–0.79)	0.040
Parental hip fracture	0.79 (0.63–0.99)	0.27 (0.13–0.56)	0.006
Glucocorticoid use	0.76 (0.61–0.94)	0.75 (0.28–2.01)	>0.30
Smoking	0.72 (0.58–0.88)	1.93 (0.78–4.79)	0.037
Alcohol	0.76 (0.62–0.94)	0.68 (0.24–1.92)	>0.30
Rheumatoid arthritis	0.72 (0.58–0.90)	0.83 (0.40–1.70)	>0.30
Secondary osteoporosis	0.77 (0.61–0.97	0.71 (0.47–1.08)	>0.30

The *p*‐value represents significance for the interaction between presence and absence of the risk factor.

## Discussion

The SCOOP study is the first prospective, randomized study to utilize the absolute risk of fracture, determined as the FRAX 10‐year probability of hip fracture, as the means to target intervention to reduce fracture risk. It has demonstrated the effectiveness of a community‐based, screening program in women aged 70 to 85 years to reduce hip fractures, showing an average 28% reduction in incidence.^12^ Because intervention was targeted only at those women with high risk of hip fracture, we hypothesized that there would be an interaction between baseline risk and screening effectiveness, and the present analysis confirms this to be the case. In individuals at very high risk of hip fracture, the estimated reduction in hip fracture risk was more than 50%.

The study design of the SCOOP study has strengths and limitations. The pragmatic design, in which a novel strategy was directly compared with existing practice, allowed assessment of the effectiveness of the screening pathway in reducing hip fracture risk.^12^ The main limitation is the lack of BMD measurements in some of the screening arm and the vast majority of the control arm, impairing the ability to undertake traditional post hoc subgroup analyses. However, the use of FRAX probabilities as a continuous variable has been used to investigate interactions with treatment effects in a number of previous analyses,^4^, ^5^, ^6^, ^7^, ^8^, ^9^, ^10^, ^11^ and it is particularly apt for use in the current study where the exposure to treatment varied by the baseline risk. The greater reduction in hip fracture risk at higher baseline risk suggests that treatment rather than other factors explained the observed effect.

Exploratory examination of individual FRAX risk factors shows that the screening effectiveness was particularly influenced by the presence of the two most prevalent clinical risk factors in the high‐risk group, namely prior fracture and parental history of hip fracture. For both risk factors, the effect of screening was greater in those with the risk factor present, raising questions about the potential underlying mechanism. The most obvious and most likely mechanism, of course, is that these two factors drove the increased exposure to treatment in the intervention arm. The well‐documented association between prior fracture and lower BMD might suggest that the greater effect was additionally mediated by the presence of a lower or osteoporotic BMD, but the evidence base for that is questionable for most antiresorptive medications.^15^ The effective reduction in hip fracture risk in these 70‐ to 85‐year‐old women with prior fracture is, however, supportive of the many international and national guidelines, including those in the UK, which state that all such women should be strongly considered for osteoporosis therapy, without necessarily measuring BMD, recently recognized as an unmet need in closing the osteoporosis treatment gap.^1^, ^3^, ^16^, ^17^ There is certainly little evidence for a strong relationship between a parental history of hip fracture and BMD; indeed, meta‐analysis shows that the effect of parental history on fracture risk is almost completely independent of BMD.^18^ A further potential explanation is that both factors may have influenced persistence as well as uptake of osteoporosis therapies, a hypothesis that will be explored in a subsequent analysis of the SCOOP study. Certainly, the study participants represented motivated volunteers with a higher level of education, more fracture risk factors but less frequent smoking exposure than non‐participants.^12^

The results of the SCOOP study have potential impacts on future health care policy, including the implementation of a screening strategy for fracture prevention. Indeed, a screening approach was recommended by NICE in 2012, when it proposed that all women aged 65 years or older and men aged 75 years or older should have a fracture risk assessment using the FRAX or QFracture tools.^19^ In contrast to FRAX, the risk identified by the QFracture tool has not been examined for reversibility, intervention thresholds have not been defined, and QFracture does not permit the incorporation of BMD values into the risk assessment. The SCOOP study readily demonstrates the reversibility of high risk identified by FRAX; interestingly, the intervention thresholds used in SCOOP, defined before the launch of the first NOGG guidance in 2008, ranged from a FRAX 10‐year probability of hip fracture from 5.24% in 70‐ to 75‐year‐olds to 8.99% in 85‐year‐olds. In the recently updated NOGG guidance, treatment is recommended in patients aged 70 years or older with a 10‐year major osteoporotic fracture probability of at least 20% or a hip fracture probability of at least 5%,^3^ thresholds that are associated with very acceptable cost‐effectiveness as concluded by the recent NICE HTA on oral and intravenous bisphosphonates.^20^

Preliminary health economic analyses of the SCOOP study indicate that the cost per prevented hip fracture is less than £8000 and that the cost per quality‐adjusted life year (QALY) gained, estimated under a number of different scenarios, is less than £20,000.^12^ If one estimates that had screening been applied to the whole SCOOP study, then approximately 108 hip fractures would have been prevented. The original target population for the study comprised 52,033 women aged 70 to 85 years, suggesting that if the SCOOP strategy was applied across the whole population of 70‐ to 85‐year‐old women in the UK, estimated at 3.7 million (2016, mid‐year estimate),^21^ then almost 8000 hip fractures could be prevented each year; this could be further enhanced by mechanisms that extended the strategy to the two‐thirds of eligible women who did not participate in the screening study, as well as combining osteoporosis treatment with falls prevention in eligible individuals.

In conclusion, the analysis demonstrates an interaction between baseline FRAX hip fracture probability and a subsequent reduction in hip fracture incidence in those at higher risk targeted for appropriate treatment. Treatment success appears to have been driven by factors that might influence treatment adherence, a hypothesis that can be investigated in subsequent analyses. Future studies should examine how this FRAX‐based approach can be made available to, or accessible by, the wider community to achieve greater reductions in the number of hip fractures in the UK and elsewhere.

## Disclosures

Professor N Harvey has received consultancy, lecture fees and honoraria from Alliance for Better Bone Health, AMGEN, MSD, Eli Lilly, Servier, Shire, UCB, Consilient Healthcare and Internis Pharma. Professor McCloskey has been, or currently is, an advisor or speaker for ActiveSignal, Amgen, AstraZeneca, Consilient Healthcare, GSK, Hologic, Internis, Lilly, Medtronic, Merck, Novartis, Pfizer, Roche, Sanofi‐Aventis, Servier, Synexus, Tethys, UCB, Warner Chilcott. He has also received research support from these plus I3 Innovus, the IOF and Unilever. Professor Kanis has held grants from Amgen, Lilly, Unigene and Radius Health; non‐financial support from Medimaps, Asahi and AgNovos; Professor Kanis is the architect of FRAX but has no financial interest. Professor Cooper Cooper has received consultancy fees and honoraria from Amgen, Danone, Eli Lilly, GSK, Medtronic, Merck, Nestle, Novartis, Pfizer, Roche, Servier, Shire, Takeda and UCB. No other declarations of interest are reported.
